# Epigenetics in Prader-Willi Syndrome

**DOI:** 10.3389/fgene.2021.624581

**Published:** 2021-02-15

**Authors:** Aron Judd P. Mendiola, Janine M. LaSalle

**Affiliations:** Department of Medical Microbiology and Immunology, Genome Center, MIND Institute, University of California, Davis, Davis, CA, United States

**Keywords:** epigenetic, imprinting, neurodevelopment, metabolic, circadian, diurnal, genetic, obesity

## Abstract

Prader-Willi Syndrome (PWS) is a rare neurodevelopmental disorder that affects approximately 1 in 20,000 individuals worldwide. Symptom progression in PWS is classically characterized by two nutritional stages. Stage 1 is hypotonia characterized by poor muscle tone that leads to poor feeding behavior causing failure to thrive in early neonatal life. Stage 2 is followed by the development of extreme hyperphagia, also known as insatiable eating and fixation on food that often leads to obesity in early childhood. Other major features of PWS include obsessive-compulsive and hoarding behaviors, intellectual disability, and sleep abnormalities. PWS is genetic disorder mapping to imprinted 15q11.2-q13.3 locus, specifically at the paternally expressed *SNORD116* locus of small nucleolar RNAs and noncoding host gene transcripts. *SNORD116* is processed into several noncoding components and is hypothesized to orchestrate diurnal changes in metabolism through epigenetics, according to functional studies. Here, we review the current status of epigenetic mechanisms in PWS, with an emphasis on an emerging role for *SNORD116* in circadian and sleep phenotypes. We also summarize current ongoing therapeutic strategies, as well as potential implications for more common human metabolic and psychiatric disorders.

## Introduction

### Clinical Features and Metabolic Phases of PWS

Prader-Willi Syndrome (PWS) is initially characterized by infantile hypotonia, failure to thrive due to poor suck, small hands and feet, and hypogonadism due to growth hormone deficiencies (Holm et al., 1993; Cassidy et al., 2012; Butler, 2020). During childhood, the development of extreme hyperphagia leads to obesity if not controlled is a major clinical feature of PWS. Other PWS features include obsessive-compulsive disorders, behavioral difficulties, intellectual disability, and sleep abnormalities.

PWS clinical characteristics are classically divided into two nutritional stages; however, it was recently identified that the stages are more complex and can be subdivided into five stages as described in Table 1 (Miller et al., 2011; Butler et al., 2019b). The first stage (phase 0) occurs *in utero*, characterized by decreased movement in the womb and a low birth weight and size. Generally undiagnosed until birth, infants are assessed for PWS through a series of physical tests that determine the state of reflex and musculature (Holm et al., 1993; Miller et al., 2011; Cassidy et al., 2012). The next stage (phase 1a) of PWS is characterized by hypotonia, which leads to poor feeding and a resultant failure to thrive. Eventually, feeding normalizes entering phase 1b, but difficulty in feeding remains, and PWS infants often lag in meeting standard developmental milestones. In the more severe cases of PWS, cranial and skeletal features are also apparent (Kindler et al., 2015). Although development is altered and delayed at infancy, patients feeding normalizes resulting in a steady increase in weight. However, stage 2 of nutritional development persists through early childhood, characterized by extreme fixation on food and development of hyperphagia (Holm et al., 1993; Cassidy and Driscoll, 2009; Miller et al., 2011). Stage 2 is divided into two phases in which phase 2a is an increase in weight that occurs without changes in appetite or feeding followed by phase 2b, characterized by fixation on food leading to phase 3, hyperphagia. In PWS, hyperphagia is developed at 2 years of age on average, and the severity of hyperphagia varies between children (Miller et al., 2011; Kim et al., 2012; Relkovic and Isles, 2013). Food intake and presence can be controlled by caretakers through proper rationing, reinforcement, and care which is most effective in the early PWS nutritional stages. However, hyperphagia continues to be a life-long struggle that is difficult to control with mitigation efforts. As PWS enters later stages of childhood and into adolescence, some patients enter the final stage (phase 4) and are able to feel full due to increased satiety and decreased behavioral difficulties related to food. It is unclear whether all PWS patients enter phase 4. Severity of clinical features is attributed to the size of deletions and may impact the recovery from hyperphagia (Kim et al., 2012).

**Table 1:** Clinical characteristics of nutritional phases.

Phase 0	Decreased fetal movement and growth restriction	*In utero*
Phase 1a	Infant becomes hypotonic and can develop failure to thrive	~0–9 months
Phase 1b	Infant begins to feed and grows steadily along a growth curve	~9–25 months
Phase 2a	Weight increase, without significant change in appetite or caloric intake	~2–4 years of age
Phase 2b	Continuous weight gain with increased food interest	~4–8 years of age
Phase 3	Development of hyperphagia, increased food seeking, and lack of satiety	~8 years of age
Phase 4	Loss of insatiable appetite and can feel full	Adulthood

Miller et al. (2011) and Butler et al. (2019b).

Although abnormal sleep patterns are not featured in the nutritional PWS stages, disrupted REM sleep is a severe clinical feature in PWS. Patients with PWS exhibit a disrupted sleep pattern, which is similar to narcolepsy, including increased daytime sleepiness coupled to alterations to REM sleep at night. It is possible that the disrupted REM sleep is directly linked to the other clinical features in PWS. The importance of sleep is critical to the establishment of epigenetic patterns that solidify a diurnal pattern of feeding and metabolism. Once established, this diurnal rhythm is responsible for timing mechanisms regulating development from infancy through adulthood. Disruption of these rhythmic patterns may be causing the delay in development, resulting in the PWS clinical features including hyperphagia, inability to communicate, intellectual disabilities, behavioral difficulties, and obsessive-compulsive tendencies. Abnormal sleep patterns have been well-established in PWS, however, the molecular outcomes and downstream effects are not well understood. In this article, we will review what is known, delve into promising research findings, as well as discuss some therapeutic strategies for PWS that are either encouraging or controversial.

### Molecular Genetics of PWS

**Figure 1:**
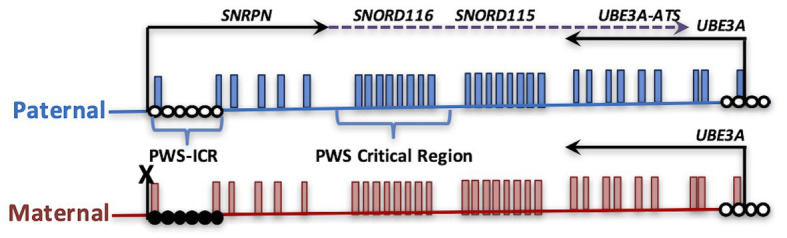
Parental imprinting in the heart of the Prader-Willi syndrome (PWS) locus. The PWS on human chromosome 15q11.2-q13.3 is shown, depicting transcripts specifically expressed from the paternal (blue) or maternal (red) alleles. PWS patients with rare paternal microdeletions have defined the critical region over *SNORD116*. DNA methylation (closed circles) on the maternal allele of the PWS imprinting control region (PWS-ICR) silences the expression of *SNRPN* (solid arrow) and the long noncoding transcript expressed in neurons (dotted arrow) that encompasses repeated snoRNA clusters (including *SNORD116* and *SNORD115*) and the antisense transcript to *UBE3A* (*UBE3A-ATS*). *UBE3A* encodes an E3 ubiquitin ligase protein that regulates protein turnover of multiple cytoplasmic and nuclear factors. Since the paternal *UBE3A* allele is silenced by the expression of *UBE3A-ATS* in neurons, deletion or mutation of the maternal copy of *UBE3A* causes Angelman syndrome.

PWS is both a genetic and epigenetic disorder, mapping the imprinted chromosomal domain of 15q11.2-13.3. Common to all cases of PWS is the absence of an expressed paternal copy of the *SNORD116* locus. Due to parental imprinting of the locus, outlined in more detail in the next section, loss of *SNORD116* can occur through deletion, uniparental disomy, or imprinting error. Most cases of PWS are caused by a large 6 Mb deletion of the entire 15q11.2-q13.3 locus (Holm et al., 1993; Cassidy et al., 2012). Two major large deletion classes include those with breakpoints at BP1 vs. BP2 combined with the downstream BP3 common deletion (Butler, 2020). However, microdeletions of the imprinting control region upstream of *SNRPN* (Figure 1) also result in loss of expression of *SNORD116* due to loss of the promoter. Rare microdeletions that only encompass *SNORD116*, but not *SNRPN* or *SNORD115*, have also been found in patients with PWS (Sahoo et al., 2008; de Smith et al., 2009; Duker et al., 2010). Approximately 60% of patients have paternal deletions, 36% are a result of maternal uniparental disomy, 4% are due to imprinting mutations that lead to a maternal imprinting status, and <1% are microdeletions of *SNORD116* (Butler et al., 2019a). What is common to all causes of PWS is the absence of *SNORD116* expression (Sahoo et al., 2008; Duker et al., 2010; Bieth et al., 2015; Rozhdestvensky et al., 2016).

While these findings establish that the lack of paternally expressed *SNORD116* is the likely predominant cause of PWS, there are a greater number of genes in the locus that may contribute to phenotypes of PWS. Both PWS large deletions include *MRKN3*, *MAGEL2*, *NDN*, *NPAP1*, *SNRPN*, *SNORD repeats*, *UBE3A*, *ATP10A*, *GABRB3*, *GABRA5*, *GABRG3*, *OCA2*, and *HERC2.* Additional genes between the proximal 15q11.2 breakpoints BP1 and BP2 include *TUBGCP5*, *CYFIP1*, *NIPA1*, and *NIPA2*. Genotype-phenotype investigations between the major molecular subtypes have been somewhat revealing at improving understanding of the genes involved in specific PWS phenotypes. In deletion compared to non-deletion etiologies of PWS, sleep abnormalities were more common (Torrado et al., 2007). Adaptive behavior scores were worse in PWS individuals with BP1-BP3 compared to BP2-BP3 or UPD and obsessive-compulsive behaviors more common in BP1-BP3 compared to UPD (Butler et al., 2004). In the Reiss Screen for maladaptive behaviors, deletion PWS patients showed higher self-injury and stealing scores compared to UPD (Hartley et al., 2005). Together, these studies indicate that gene expression patterns of one or more of these genes may contribute to variable phenotypes within PWS between the molecular subclasses. Below, the imprinted genes in the locus that have been implicated in PWS phenotypes will be discussed in more detail, as well as the cluster of biallelically expressed GABA_A_ receptor genes (*GABRB3*, *GABRA5*, and *GABRG3*), which are implicated in some of the neuropsychiatric phenotypes that are more severe in the deletion PWS molecular subclass.

*SNORD116* is processed through a long noncoding transcript that initiates at the imprinting control region upstream of *SNRPN*, followed by two repeat clusters of small nucleolar RNAs (snoRNAs *SNORD116* and *SNORD115*) and terminating at the *UBE3A* antisense transcript (Figure 1; Sutcliffe et al., 1994; Buiting et al., 1995; Runte et al., 2001; Landers et al., 2004; Vitali et al., 2010; Chamberlain, 2013). In humans, *SNORD115*, but not *SNORD116* or *UBE3A-ATS*, is exclusively expressed in neurons, while *Snord116*, *Snord115*, and *Ube3a-ats* are all neuron-specific transcripts in mouse. *SNORD115* and *SNORD116* encompass clusters of repeated subunits of sequences encoding a C/D box snoRNAs embedded within intronic regions of the noncoding exons encoding the snoRNA host transcript *SNHG14* (Cavaillé et al., 2000; de los Santos et al., 2000; Bortolin-Cavaillé and Cavaillé, 2012; Stanurova et al., 2018). C/D box snoRNAs have known functions in regulating 2-O methylation rRNA modifications by recruiting ribonucleoprotein complexes including fibrillarin, which catalyzes methylation (Dupuis-Sandoval et al., 2015; Bratkovič et al., 2020).

**Figure 2:**
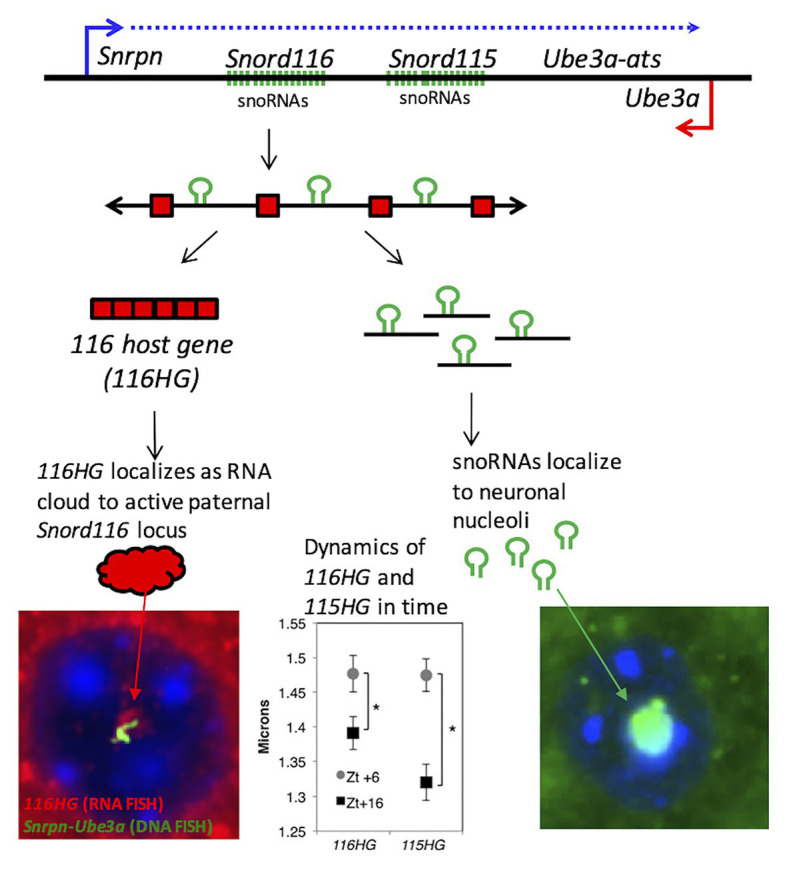
PWS noncoding RNA summary. **(Top panel)** Individual components of the processed PWS snoRNA-lncRNA region between *Snrpn* and *Ube3a*. Within the *Snord116* and *Snord115* loci are repeated units of snoRNAs (green), lncRNA exons (red boxes), and introns with G-C skew. Processing results in spliced *116HG* and *115HG* lncRNAs that localize to their sites of transcription, the snoRNAs that localize to nucleoli, and R-loops that displace histones and promote locus chromatin decondensation. **(Bottom left panel)** Seen by RNA-FISH, *116HG* forms a large RNA cloud (red) localized to the decondensed paternal allele (green) in nuclei (blue), associated with 2,403 genes enriched for metabolic function. *116HG* and *115HG* RNA clouds are significantly larger at diurnal time ZT6 (sleep) than ZT16 (wake), corresponding to gene dysregulation in *Snord116^+/−^* specifically at ZT6. **(Bottom right panel)** Processed *Snord116* snoRNAs (green) localize to a single nucleolus in mature cortical neurons.

SnoRNAs are processed from introns of the *SNORD116* and *SNORD115* within the *SNHG14* host gene subunits, called as *116HG* and *115HG* (Figure 2; Cavaillé et al., 2000; Leung et al., 2009; Vitali et al., 2010). Unlike other C/D box snoRNAs, *SNORD116* and *SNORD115* are classified as “orphan snoRNAs” because their targets and functions are unknown (Bratkovič et al., 2020). Previous studies have shown that *SNORD116* localizes in the nucleolus and may participate in splicing and RNA modifications (Bazeley et al., 2008; Leung et al., 2009). In contrast, *116HG* and *115HG* localize in the form of RNA “clouds” at the site of their own transcription in the nucleus (Figure 2), and dynamically regulate many additional genes across the genome (Powell et al., 2013; Coulson et al., 2018b). *SNORD115* is also shown to be involved in the alternative splicing specifically of the serotonin receptor *5-HT2C* mRNA (Bazeley et al., 2008; Raabe et al., 2019). Although, both loci are potentially implicated in PWS, microdeletion of only the *SNORD115* cluster does not lead to the PWS phenotype in humans (Runte et al., 2005). To date, the precise mechanisms of how *Snord116* functions are critical for neurodevelopment remain elusive, however, advancements in sequencing technology have provided new insights and will be covered in more detail in the section below. In addition to *SNORD116*, other genes in the 15q11.2-13.3 locus, including *NECDIN*, *MAGEL2*, and a cluster of GABA receptor genes are implicated in the phenotypes observed in most cases of PWS.

*NECDIN* (*NDN*) is an imprinted gene that is paternally expressed and encodes for the protein NECDIN, which belongs to the melanoma antigen-encoding gene (*MAGE*) family of proteins that are enriched in differentiated cells. *NDN* is one of several protein coding genes deleted from the large 6 Mb chromosomal deletion observed in PWS patients and is implicated in neuronal maturation (Ren et al., 2003). Other than its role in cellular differentiation and neuronal maturation, *NDN* is also involved in neurite and axonal growth, arborization, migration, and fasciculation, which are important for normal neurological signaling and development (MacDonald and Wevrick, 1997; Kuwajima et al., 2006; Davies et al., 2008; Miller et al., 2009; Bervini and Herzog, 2013). Mouse models of *Ndn* deficiency have been instrumental for studying abnormal brain development and cognitive impairments in PWS. However, studies using *Ndn* deficient mice did not exhibit any morphological differences in brain development, but led to respiratory failure causing apneas and irregular breathing patterns that are caused by increased activity in serotonin transporter (SERT/slc6a4; Matarazzo et al., 2017). Furthermore, *Ndn* knockout mice exhibit a higher pain threshold due to a decrease in nerve growth factor sensory neurons (Kuwako et al., 2005). Respiratory failure and higher pain thresholds are also observed in patients with PWS (Rittinger, 2001; Butler et al., 2002; Angulo et al., 2015). Specifically, irregularities in breathing may be a large proponent to sleep abnormalities in PWS.

*MAGEL2* is another imprinted gene, that is, paternally expressed and encodes for the protein MAGEL2 that belongs to the MAGE family of proteins. Truncated *MAGEL2* mutations cause PWS-like phenotypes observed in patients (Schaaf et al., 2013; Fountain and Schaaf, 2016), but these cases have been recently distinguished from PWS in a new classification of Schaaf-Yang syndrome (SYS). SYS shares phenotypic overlap with PWS, but also exhibit distinct behavioral and metabolic phenotypes including autism spectrum disorder (Fountain and Schaaf, 2016). In mouse embryogenesis, *Magel2* is highly expressed in non-neuronal (placenta, midgut turbucle, and midgut region) and neuronal tissue types (dorsal root ganglia and peripheral neurons surrounding limb and trunk muscles (Bervini and Herzog, 2013). In adult mouse brain, *Magel2* is highly enriched in hypothalamic regions and extends to the superchiasmic nucleus, specific regions that regulate feeding and circadian rhythms, respectively (Kozlov et al., 2007; Mercer et al., 2009). The prevalence of *MAGEL2* in the hypothalamus initially identified it as strong candidate for the hyperphagia phenotype of PWS. However, SYS patients and mouse models with *MAGEL2* mutations show a lower prevalence of overeating and obesity. Instead, it was determined that *MAGEL2* functions as a ubiquitin transporter that localizes in SCN neurons and acts as a direct regulator of circadian clock proteins through ubiquitination (Mercer et al., 2009; Tacer and Potts, 2017; Vanessa Carias et al., 2020).

The 15q11-q13 PWS region also contains a cluster of three genes encoding subunits of receptors for the neurotransmitter, GABA_A_. GABA is the major inhibitory neurotransmitter in the postnatal brain, so loss of these GABA receptors in the large deletion cases of PWS is expected to be involved in some of the phenotypes of PWS. 15q11.2-13.3 genes *GABRB3*, *GABRA5*, and *GABRG3* encode for β3, α5, and γ3 subunits, respectively. GABA_A_ receptors are assembled into hexameric protein complexes made up of combinations of a1–6, b1–3, g1–3, and other subunits, with α5 containing receptors making up ~5% of GABA_A_ receptors in human brain (Mohamad and Has, 2019). Unlike the imprinted genes in the PWS locus, these 15q11.2-13.3 GABA_A_ receptor genes are biallelically expressed in the brain. However, monoallelic expression and decreased protein expression of each GABA_A_ receptor subunits have been observed in autism postmortem brain (Samaco et al., 2005; Hogart et al., 2007). Furthermore, both transcript and protein levels of GABRB3 were not correlated with copy number in an analysis of PWS, AS, and 15q11.2-13.3 duplication syndrome postmortem brain (Scoles et al., 2011). A recent study on phenotypes and gene expression patterns in a *Gabrb3* deletion mouse model is also consistent with complex gene regulation, as neighboring *Oca2* expression was reduced and ocular hypopigmentation observed (Delahanty et al., 2016). Dysregulated gene expression of the 15q11.2-13.3 GABA_A_ receptors is expected to have consequences for the balance of inhibitory and excitatory signals that regulate sleep, metabolism, and mood in PWS. Recently, it has been shown that levels of GABA metabolites vary between different molecular subclasses of PWS (Lucignani et al., 2004; Rice et al., 2016; Brancaccio et al., 2017). Since there are major targets for therapeutic intervention in multiple neurodevelopmental disorders, understanding their altered expression in PWS is expected to be important for the treatment of other neurodevelopmental disorders (Braat and Kooy, 2015).

## Epigenetic Mechanisms in PWS

### Epigenetic Regulation of the Imprinting Control Region in PWS

As mentioned in the previous section on molecular genetics, small deletions of the imprinting control region (PWS-ICR) are sufficient to cause PWS when inherited on the paternal allele. Interestingly, the ICR at 15q11.2-13.1 is actually bipartite, because maternal microdeletions of a region called as the AS-ICR are found in rare cases of Angelman syndrome (Buiting et al., 1995; Smith et al., 2011). Subsequent studies in a variety of mammals have demonstrated that the AS-ICR contains alternate 5' noncoding exon for *SNRPN* that are uniquely expressed in oocytes, but not sperm or other tissues (Smith et al., 2011; Lewis et al., 2015, 2019). It is the oocyte-specific transcription that leads to methylation and transcriptional silencing of the maternal allele specifically on the maternal but not the paternal allele of the PWS-ICR. A more recent study of individuals with AS imprinting mutations have identified a more common haplotype that deletes a binding site for the transcription factor SOX2 (Beygo et al., 2020). Together, these studies have demonstrated that this upstream region, defined as the AS-ICR, is critical for establishing silencing of the maternal allele of the imprinted genes within the PWS locus.

In addition to being characterized by allele-specific DNA methylation, several additional epigenetic marks are differential by parental origin at the PWS-ICR. Specifically, the histone H3 lysine 9 (H3K9) methyltransferase SETDB1 associates with the transcription factor ZNF274 bound to sites within the 5' cluster of SNORD116 repeats, resulting in the deposition of maternal-specific H3K9me3 marks (Cruvinel et al., 2014). Knockdown or inhibition of either SETDB1 or ZNF274 was sufficient to induce a low level of *SNORD116* transcript expression from the normally silent maternal allele (Cruvinel et al., 2014; Wu et al., 2019; Langouët et al., 2020). Together, these results suggest some promise for possible epigenetic therapies that will be discussed at the end of this review.

### Epigenetics and Imprinting in PWS and Related Human Neurodevelopmental Disorders

In addition to PWS, loss of imprinting is involved in related neurodevelopmental disorders: Angelman (AS), 15q duplication (Dup15q), Kagami-Ogata (KOS14), and Temple (TS14) syndromes (Schanen, 2006; Kagami et al., 2015; Briggs et al., 2016). Unlike the default state of biallelic expression, imprinted genes are selectively silenced on either the maternal or paternal allele by epigenetic differences including DNA methylation and repressive chromatin modifications. Imprinted genes are clustered in discrete chromosomal loci and are regulated by a central imprinting control region (ICR), such as the PWS-ICR, in which methylation is diagnostic for AS, PWS, and Dup15q disorders (Figure 1). Some imprinted genes exhibit tissue-specific or developmental-specific imprinting patterns regulated by long noncoding RNAs. Furthermore, the largest conserved cluster of microRNA (miRNA) in the mammalian genome is found within the KOS14 imprinted locus and is responsible for regulating neuronal maturation and mTOR growth pathways (Winter, 2015). Experimental evidence is emerging for regulatory cross-talk between different imprinted gene loci (Stelzer et al., 2014; Jung and Nolta, 2016; Martinet et al., 2016; Vincent et al., 2016; Lopez et al., 2017), but this emerging “imprinted gene network” hypothesis (Fauque et al., 2010; Haga and Phinney, 2012; Monnier et al., 2013; Ribarska et al., 2014) has been understudied in the context of the developing nervous system.

RNA FISH has shown that *116HG* localizes in the nucleus, where it forms an RNA cloud that is absent in *Snord116* deletion brain. *116HG* was also found to colocalize with metabolic, circadian, and epigenetic gene loci including *Mtor*, *Clock*, *Cry1/2*, *Per1/2/3*, *Dnmt1/3b*, *Tet1/2/3*, *Mecp2*, and others at ZT6, the time point with the largest effect of *Snord116* deletion on transcription globally (Powell et al., 2013). *Snord116*’s involvement in transcriptional regulation, therefore, prompted an investigation of epigenetic differences that may explain the interaction of *Snord116* with diurnal light cycles. Whole genome bisulfite sequencing (WGBS) was performed on cortex samples from wild-type (WT) and PWS mice sacrificed every 3 h starting from Zt0–Zt16 and showed that *Snord116* is involved in regulating a dynamic rhythm of diurnal methylation (Coulson et al., 2018b). Rhythmically methylated CpG dinucleotides were identified (<1% of all CpGs) within enhancers and promoters of genes that were undergoing a pattern of reduced methylation during sleep (light hours) in wild-type mouse cortex, a pattern that was lost upon *Snord116* deletion. The differentially methylated regions mapped to genes involved in circadian rhythms, metabolism, and epigenetic regulation, similar to the prior genes identified associated with *116HG*. Table 2 gives examples of specific genes in each of these categories that were identified by multiple unbiased genomic approaches in both studies. A large portion of genes identified are involved in adding, removing, and recognizing DNA methylation while other genes are important transcriptional regulators for development. Further integration of promoter methylation and RNA-seq data revealed that genes being diurnally dysregulated were central to the body weight, behavior, and metabolic phenotypes of PWS (Coulson et al., 2018b).

**Table 2:** Examples of *Snord116* associated and impacted genes and predicted functions.

Category	Function	Gene name	Gene binding to *116HG*^a^	*Snord116*-dependent transcriptional change^a^	*Snord116*-dependent DNA methylation change^b^	
					Mouse	Human
Epigenetic	Methyl binding protein critical to neurodevelopment	*Mecp2*	Yes	Increased at Zt6	No	No
	Binds DNA:RNA hybrids	*Setx*	No	Increased at Zt6	Yes	Yes
	DNA demethylases	*Tet1*	No	Increased at Zt6	Yes	Yes
		*Tet2*	No	Increased at Zt6	Yes	No
		*Tet3*	No	Increased at Zt6	Yes	Yes
	Histone deacetylases	*Hdac3*	No	Increased at Zt6	No	No
		*Hdac4*	No	Increased at Zt6	Yes	Yes
		*Hdac5*	No	Increased at Zt6	Yes	Yes
	DNA methyltransferases	*Dnmt1*	No	Increased at Zt6	No	Yes
		*Dnmt3a*	Yes	Increased at Zt6	No	Yes
Circadian	Establishes phases and periods	*Per2*	No	Increased at Zt6	No	Yes
		*Per3*	No	Increased at Zt6	No	No
		*Arntl*	Yes	Increased at Zt6	Yes	Yes
Metabolic	Kinase involved in regulating cellular energy homeostasis	*Mtor*	Yes	Increased at Zt6	No	Yes
Transcription	Transcriptional regulator of E-box motif containing genes	*Neurod1*	No	Increased at Zt6 & Zt16	Yes	No

a Full gene lists are included in Powell et al. (2013).

b Full gene lists are included in Coulson et al. (2018b).

The Coulson et al. study also demonstrated a molecular connection between the *116HG* and the KOS14 locus, building upon a prior study showing a connection between *IPW* (part of the *116HG* transcript) and *DLK1* regulation at the KOS14/TS14 locus in human neuronal culture (Stelzer et al., 2014). In this case, DNA FISH was used to examine chromosome decondensation, a measurement of neuronal activation of the paternal allele resulting from histone displacement, at both PWS and TS14 loci in adult mouse brain at six different diurnal time points. Interestingly, the TS14 locus only showed evidence of active chromatin decondensation in *Snord116* deletion mouse cortex. Furthermore, chromatin decondensation at the PWS locus did occur in *Snord116* deletion, but the timing was shifted from light to dark cycle, similar to the effects observed on DNA methylation. Together, these results suggest that the ancestrally older imprinted TS14/KOS14 locus may become more active as a compensatory mechanism to fill in for loss of *Snord116*, but this comes at a cost of proper timing of these epigenetic events.

### Epigenetics and Imprinting of Mammalian Imprinted Loci and the Emerging Importance in Circadian Rhythmicity and Sleep

Daily and seasonal cycles of light, temperature, and feeding govern energy and activity of organisms from all branches of life. These environmental and metabolic inputs play an important role in the synchronization of the core circadian clock with the rhythmic patterns of many physiological and behavioral processes in peripheral tissues (Wright et al., 2013; Legates et al., 2014; Mukherji et al., 2015; Blasiak et al., 2017). The genetically encoded circadian cycle and the environmentally regulated diurnal/nocturnal cycle are integrated by a complex regulatory feedback network, which acts at the chromatin, transcriptional, and translational levels to coordinate biological and environmental rhythms (Koike et al., 2012; Papazyan et al., 2016; Takahashi, 2017). In mammals, the core circadian clock resides in the suprachiasmatic nucleus of the hypothalamus; however, almost half of all transcripts, both protein-coding and non-coding, exhibit diurnal rhythms in one or more peripheral tissues (Yan et al., 2008; Zhang R. et al., 2014). While most studies on circadian biology focus on the suprachiasmatic nucleus, investigations into diurnal rhythms of cerebral cortex are relevant to the cognitive deficits in PWS and to energy expenditure. For instance, circadian and metabolic genes showed light-cycle-specific dysregulation in the *Snord116del* mouse model, corresponding to cyclical dynamics of *Snord116* expression (Powell et al., 2013). Rhythmic epigenetic dynamics within the cerebral cortex are less well characterized; however, increasing evidence indicates a role for DNA methylation in these rhythms. Approximately 6% (25,476) of CpG sites assayed by 450k array are dynamically regulated throughout diurnal and seasonal cycles in human cortex (Lim et al., 2017). This epigenetic plasticity plays an important role in circadian entrainment and the resiliency of the circadian clock to changes in the diurnal environment (Stevenson and Prendergast, 2013; Azzi et al., 2014; Lim et al., 2014).

The 14q32.2 imprinted locus bears striking similarity to the PWS locus, as it encodes the only other repetitive cluster of snoRNAs in the mammalian genome (*SNORD113* and *SNORD114*), which are maternally expressed and exhibit allele-specific chromatin decondensation in neurons, similar to *SNORD116* and *SNORD115* (Cavaillé et al., 2002; Tierling et al., 2006; Leung et al., 2009). TS and KOS are reciprocally imprinted disorders, with TS caused by maternal uniparental disomy 14 [UPD(14)mat], and KOS caused by paternal uniparental disomy 14 [UPD(14)pat]. Loss of paternal gene expression at this locus in TS, results in aberrantly high expression of maternal non-coding RNAs, including *SNORD113* and *SNORD114*, whereas KOS results from the loss of maternally expressed, non-coding RNAs and the upregulation of paternally expressed *DLK1*. Interestingly, TS phenocopies PWS suggesting that these two imprinted loci may perform similar functions and share common pathways (Temple et al., 1991; Hosoki et al., 2009; Kagami et al., 2015). The loss of *Snord116* in PWS increases gene expression in the TS locus, indicating that the two loci may interact through a cross-regulatory network. In support of this hypothesis, *IPW* from the PWS locus has been shown to regulate the TS locus in an induced pluripotent stem cell line of PWS (Stelzer et al., 2014). Though both PWS and TS loci show circadian oscillations, the mechanism of this regulation and the impact of circadian rhythms on their cross-regulation suggests that a balance between the two loci is critical for sleep and metabolism (Labialle et al., 2008a,b; Powell et al., 2013).

**Figure 3:**
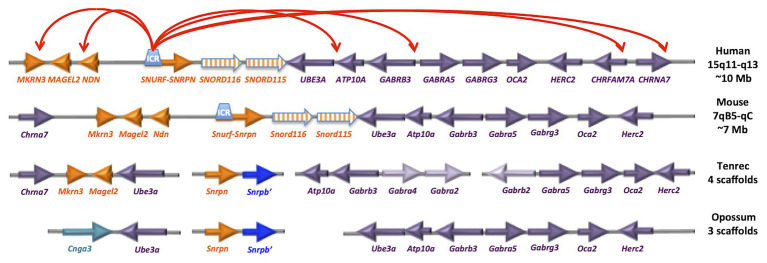
The PWS/AS imprinted locus has emerged recently within placental mammals. The gene orientation and linear organization is shown for human and mouse, as well as the earliest placental mammal (tenrec) and marsupial (opossum). The red arrows on top represent results from neuronal 4C analysis of chromatin looping (Yasui et al., 2011). Interestingly, the tenrec arrangement of *Chrna7-Mkrn3-Magel2-Ube3a* (spanning ~500 kb) is similar to the human 4C long-range interactions spanning ~10 Mb, despite the lack of evidence for *Snrpn* or *Snord* clusters at the locus. Humans (and chimps) have 22 *SNORD116* and 44 *SNORD115* copies, mouse has 27 detectable *Snord116* and 130 *Snord115* copies.

Most imprinted loci, such as *IGF2*, *PEG1/MEST*, and *IGR2R*, are imprinted in marsupials as well as eutherian (placental) mammals (Figure 3). In contrast, *Snrpn* and *Ube3a* are not imprinted in marsupials and are on distinct chromosomes (Rapkins et al., 2006). Interestingly, the ancestral eutherian mammal tenrec (*Echinops telfairi*) lacks the *Snord116* and *Snord115* genes and *Snrpn* and *Ube3a* are on separate chromosomes (Rapkins et al., 2006; Yasui et al., 2011; Zhang Y. J. et al., 2014). Humans (and chimps) have 22 *SNORD116* and 44 *SNORD115* copies, while mouse has 27 detectable *Snord116* and 130 *Snord115* copies. Potentially relevant for the PWS phenotype, tenrecs have a unique metabolic and sleep structure among mammals adapted to long periods of reduced activity and body temperature called torpor (Lovegrove and Génin, 2008; Lovegrove et al., 2014a,b). Non-REM sleep and periods of torpor are thought to be ancestrally adaptive to conserve energy and escape predation. Eutherian mammals have distinct adaptations for daily sleep and activity patterns based on diet, body size, and brain size (Siegel, 2005; Gerhart-Hines and Lazar, 2015).

Unlike the PWS/AS locus, the chromosomal arrangement of the *SNORD113*/*SNORD114* cluster at the KOS/TS locus is similar in monotremes, marsupials, and placental mammals, and the miRNAs at this cluster are evolutionarily stable (Zhang Y. J. et al., 2014). Both imprinted snoRNA loci exhibit neuron-specific chromatin decondensation (Leung et al., 2009) and also show evidence for diurnally expressed transcripts, many of which are also dysregulated in *Snord116del* mice (Powell et al., 2013; Coulson et al., 2018b). Interestingly, circadian rhythmicity of the *Dlk1/Dio3* (Labialle et al., 2008a,b) and *Magel2* (Kozlov et al., 2007; Devos et al., 2011; Tennese and Wevrick, 2011) loci and cross-regulation between PWS/AS and *DLK1* loci have been described previously (Stelzer et al., 2014) but are poorly understood at a mechanistic level.

Despite its function being fully known, loss of *Snord116* in PWS mouse models has been demonstrated to dysregulate sleep, feeding, and temperature cycles (Lassi et al., 2016a,b). These studies have demonstrated the importance of hypothalamic *Snord116* expression on temporally regulated behavior. Interestingly, *Snord116* deficient mice exhibited disrupted feeding cues induced by erratic behavior due to increased activity prompted by foraging. Reminiscent of humans with PWS, *Snord116* deficient mice exhibited a strong fixation on food and high food intake irrespective of weight gain (Lassi et al., 2016a). Furthermore, *Snord116* deficient mice also exhibited a prolonged REM phase that was uncoupled with normal circadian patterning (Lassi et al., 2016b). Together, these studies have demonstrated the importance of *Snord116* on temporally regulated behaviors including sleep, feeding, foraging, and temperature regulation that are consistent with the recent evolutionary selection of the imprinted PWS locus in mammalian-specific diurnal cycles.

Multiple studies have also explored the role of *Snord116* in hypothalamic regulation of hormones linked to diurnal behaviors. Orexin neurons in the hypothalamus facilitate sleep-wake cycles by regulating hormones that promote wakefulness (noradrenaline, histamine, and acetylcholine) and rest [melanine-concentrating hormone (MCH); Pace et al., 2020]. Loss of orexin neurons are widely implicated in dysregulated sleep in patients with narcolepsy and has been observed in patients with PWS as well (Vgontzas et al., 1996; Chemelli et al., 1999; Mignot et al., 2002; Omokawa et al., 2016). However, it was not until recently that loss of *Snord116* was demonstrated to decrease orexin neuron levels in the lateral hypothalamus without altering levels of MCH and MCH neurons in mice (Pace et al., 2020). A decrease in orexin neurons may facilitate the prolonged REM sleep characteristic of PWS due to the imbalance in orexin/MCH ratio with a higher MCH concentration during wake cycles promoting more rest (Pace et al., 2020). This phenomenon is not unique to *Snord116* deletion mice, however, as this orexin/MCH imbalance was also observed in *Magel2* deficient models (Kozlov et al., 2007).

Patients with PWS are characterized as having reduced levels of growth hormone, but elevated levels of ghrelin (Tauber et al., 2019). Ghrelin is the endogenous ligand of growth hormone secretagogue receptor 1a. Ghrelin is peptide produced by the gut with a diversity of physiological effects, including appetite stimulation and lipid accumulation. Subsequent studies have demonstrated that it is actually the acylated form of ghrelin that is elevated in PWS children and young adults, while nonacylated ghrelin levels are indistinguishable from controls (Kuppens et al., 2015). However, while both growth hormone and ghrelin are known to have clear diurnal patterns of secretion, with nocturnal levels being higher than daytime levels in humans, there have been a surprising lack of investigation into the possibility growth hormone abnormalities in PWS may be due to altered diurnal rhythms (Kyung et al., 2004; Stawerska et al., 2020).

Despite orexin neurons being a critical cell type for *Snord116* regulation on hormonal regulation from the hypothalamus, loss of *Snord116* in other brain regions, such as cerebral cortex, also appear to contribute to the proper expression of core circadian clock regulators such as *Per* and *Bmal* genes (Powell et al., 2013; Coulson et al., 2018a). These findings reinforce the role of *Snord116* in establishing multiple aspects of circadian rhythms that are lost upon deletion. Studying *Snord116* and identifying its targets can contribute to the development of therapeutic interventions that target sleep and metabolism which are critical to development.

### PWS Mouse Models for Preclinical Testing of Therapeutic Interventions

Mouse models of *Snord116* deficiency that recapitulate some features of PWS have been created as useful models for testing possible therapeutic interventions. Like in humans, *Snord116* is a maternally imprinted gene in mouse and localizes to a syntenic loci chromosome 7qC. The first generation of mouse models generated were designed with large deletions mimicking those observed in humans with PWS (Yang et al., 1998). These mouse models exhibited extreme hypotonia and failure to thrive, leading to death 1 week after birth. The high lethality rate was caused by the loss of protein coding genes *Snrpn* and *Ube3a-ats*, which are hypothesized to be important to alternative splicing (Tsai et al., 1999; Bressler et al., 2001; Bervini and Herzog, 2013). As in humans with PWS, these mouse models exhibited a dysregulation of major endocrine hormones including growth hormone, glucose, and insulin, which are necessary for cellular homeostasis and proliferation. Disruption of each hormone lead to metabolic dysregulation which results in extreme hypotonia that leads to the failure to thrive.

Today, the most commonly used PWS mouse models were originally generated by two separate labs using *cre*-mediated deletion of *Snord116* (Skryabin et al., 2007; Ding et al., 2008). These mouse models were designed by a targeted insertion of *loxP* cassettes flanking the *Snord116* (Ding et al., 2008) cluster or *Snord116* and *IPW* (Skryabin et al., 2007) through homologous recombination in embryonic stem (ES) cells derived from male blastocytes. The *2-loxP* ES cells were then injected into C57Bl/6J mice that gave birth to male mice with a *2-loxP* (+/−) genotype. These mice were mated with a transgenic strain expressing *Cre* recombinase under an ovary specific promoter producing 1-loxP mice with a *Snord116* (+/−) genotype (Ding et al., 2008). For ES cells targeted with *loxP* cassettes flanking *Snord116* and *IPW*, CRE recombinase were expressed then injected into blastocytes to produce *PWScre*(+/−) (Skryabin et al., 2007). These mouse models have a 150 kb deletion of the *Snord116* cluster or a deletion that encompasses *Snord116* and *IPW*. Like previous models, both mice develop hypotonia and failure to thrive with low to no post-natal lethality. Although these mouse models do not consistently exhibit the hyperphagia phenotype, they do exhibit a significant deficiency in cognition and energy expenditure (Powell et al., 2013; Adhikari et al., 2019) making these phenotypes useful in preclinical therapeutic strategies. Furthermore, development of *2-loxp*(−/+) and *PWScre*(+/−) mice enabled the generation of several new mouse models that are able to recapitulate the hyperphagia phenotype in adult mice through *Cre*-mediated and tamoxifen induced *Snord116* deletion in the hypothalamus (Qi et al., 2016; Purtell et al., 2017; Polex-Wolf et al., 2018) and identified the disrupted REM sleep phenotypes (Lassi et al., 2016b), respectively. Previous studies have shown that *Snord116* expression in the hypothalamus is developmentally regulated and is enriched postnatally at weaning and early adulthood (Zhang et al., 2012), implicating its involvement in regulating metabolism and circadian rhythms.

### Genetic Therapies

While most genetic diseases are amenable to genetic complementation and standard gene therapy design and delivery, there are unique challenges to gene therapy in PWS because of the epigenetic and molecular complexities of the *SNORD116* locus. In the original characterization of a *Snord116* deletion mouse model of PWS, it was mentioned that a transgene containing a single snoRNA from *Snord116* was insufficient to rescue the metabolic phenotypes (Ding et al., 2008). Since it remained possible that the limitations of using either a single copy and/or an already processed snoRNA were the reason for the lack of complementation, a new transgenic mouse was created and reported by our group using the *Snord*(+/−) model (Coulson et al., 2018a). This *Snord116* transgene contained the complete subunits of *116HG* exons, introns, and snoRNAs repeated in a total of 27 copies was expressed broadly at the transcript level in all tissues, but was only spliced and processed into snoRNAs in brain. The neuron-specific splicing was attributed to the splicing factor RBFOX3, which is also known as the neuron-specific marker NeuN. In wild-type neurons, the extra copies of *Snord116* contributed to the nucleolar accumulation of processed snoRNAs as well as the size of the *116HG* RNA cloud. However, in the *Snord116* deletion PWS model, the *Snord116* transgene did not become processed or localized to these locations, indicating that an active allele was needed for correct processing and localization. In addition, the body weight phenotype of the *Snord116* mice was similar to that of the *Snord116* deletion mouse, and there was no complementation of this phenotype in the cross.

In another study, a mouse model was generated with a *5’HPRT-LoxP-Neo^R^* insertion upstream of the maternally imprinted *Snord116* using the *PWScre*(+/−) model (Rozhdestvensky et al., 2016). The cassette insertion did not affect the PWS imprinting center methylation status, but disrupted the imprinting effect enabling expression of *Snord116* from the maternal allele, a result that was not observed in WT and KO mice without the cassette. Like the Coulson et al. study in 2018, *Snord116* was expressed across all tissue types, but in this case, the body weight phenotype was rescued in KO mice with the cassette insertion. The differences in results may depend on the imprinting mechanism of the PWS region as well as the genomic location of *Snord116*. For instance, when the *Snord116* transgene is introduced outside of the imprinted region, as would be the case for most gene therapy strategies, the ability to complement the missing paternal allele is expected to be challenging. These results demonstrate the complexities of this locus and suggest that gene therapy for PWS using conventional complementation strategies will be problematic. Despite the issues, these results also highlight the importance of targeting imprinting regulation for therapeutic interventions.

### Epigenetic Therapies

In contrast to gene therapy, epigenetic therapy for PWS has a stronger potential for clinical relevance, since PWS is an inherently epigenetic disorder. The general strategy for epigenetic strategies for PWS involves de-repressing the maternal silent PWS-ICR to activate SNRPN and *Snord116* transcription (Crunkhorn, 2017; Chung et al., 2020). Recent successes using high throughput screening of small molecule libraries identified several inhibitors of EHMT2/G9a, a histone 3 lysine 9 methyltransferase, that were capable of reactivating the expression of paternally expressed *SNRPN* and *SNORD116* from the maternal chromosome, both in cultured PWS cell lines and in a PWS mouse model (Kim et al., 2017, 2019). Similarly, inhibitor of SETDB1 using shRNA knockdown resulted in partial reactivation of *SNORD116* and *116HG* in PWS-derived iPSC cell lines and neurons (Cruvinel et al., 2014). The main differences in the epigenetic changes resulting between these two epigenetic therapies was that EHMT2/G9a did not alter DNA methylation at the PWS-ICR, while SETDB1 did not show a change in H3K9me3 at the PWS-ICR. Potentially more completely, the inactivation of ZNF274 using CRISPR/Cas9 in PWS-derived iPSC lines resulted in reactivation of both *SNRPN* and *SNORD116* as well as a reduction of H3K9me3 at the PWS-ICR (Langouët et al., 2020). Together, these studies suggest that combinations of targeted epigenetic strategies for unsilencing maternal *SNORD116* hold promise for future treatments of PWS.

## Potential Developments: Relevance of *SNORD116*-Mediated Epigenetic Mechanisms Toward Common Human Diseases

While this review has focused on the relevance of epigenetic regulation of and by *SNORD116* and other genes within the locus to the pathogenesis of PWS, we expect that understanding the interactions between imprinted genes and metabolism at this locus will have relevance to other more common metabolic and neuropsychiatric human disorders. Because of the hypothalamic network alterations in PWS associated with satiety and food reward systems, this locus is considered to be a model for understanding food addictions as well as other addictive behaviors (Salles et al., 2020). The molecular mechanisms leading hyperphagia and overeating in PWS could be informative for understanding the intersections of epigenetics, diurnal rhythms, and metabolism in more common causes of overweight and obesity. Food addictions in PWS may be similar in mechanisms to those establishing other addictions. Interestingly, “morphine addiction” and “circadian entrainment” were among the gene pathway terms identified by the unbiased search for gene promoters that showed both rhythmic demethylation and increased expression during sleep in *Snord116* deletion mice (Coulson et al., 2018a), suggesting that further characterization of these pathways could be relevant to improved treatments for opioid use disorders. In addition, there are emerging links between circadian disruptions and the exacerbation of psychiatric disorders such as bipolar disorder and depression. Chronotherapy involving sleep deprivation followed by the re-entrainment of diurnal cycles has shown effectiveness in treating these common mood disorders (Gottlieb et al., 2019; D’Agostino et al., 2020).

In conclusion, the PWS locus epigenetically regulated *SNORD116* transcripts that have evolved to become parentally imprinted within mammals, in turn serve to regulate a large number of additional genes through the genome that are related to circadian rhythms, metabolic and nutritional cycles, and brain functions. Future studies designed to better understand the genomic impacts of *SNORD116* regulation is expected to have far-reaching impacts beyond the scope of PWS.

## Author Contributions

Both authors contributed to the literature review, writing, and editing of the manuscript. All authors contributed to the article and approved the submitted version.

### Conflict of Interest

The authors declare that the research was conducted in the absence of any commercial or financial relationships that could be construed as a potential conflict of interest.
